# Exome sequencing in schizophrenic patients with high levels of homozygosity identifies novel and extremely rare mutations in the GABA/glutamatergic pathways

**DOI:** 10.1371/journal.pone.0182778

**Published:** 2017-08-07

**Authors:** Edoardo Giacopuzzi, Massimo Gennarelli, Alessandra Minelli, Rita Gardella, Paolo Valsecchi, Michele Traversa, Cristian Bonvicini, Antonio Vita, Emilio Sacchetti, Chiara Magri

**Affiliations:** 1 Department of Molecular and Translational Medicine, University of Brescia, Brescia, Italy; 2 Genetic Unit, IRCCS Centro S. Giovanni di Dio Fatebenefratelli, Brescia, Italy; 3 Department of Clinical and Experimental Sciences, Neuroscience Section, University of Brescia, Brescia, Italy; 4 Department of Mental Health, Spedali Civili Hospital, Brescia, Italy; King Abdulaziz University Hospital, SAUDI ARABIA

## Abstract

Inbreeding is a known risk factor for recessive Mendelian diseases and previous studies have suggested that it could also play a role in complex disorders, such as psychiatric diseases. Recent inbreeding results in the presence of long runs of homozygosity (ROHs) along the genome, which are also defined as autozygosity regions. Genetic variants in these regions have two alleles that are identical by descent, thus increasing the odds of bearing rare recessive deleterious mutations due to a homozygous state. A recent study showed a suggestive enrichment of long ROHs in schizophrenic patients, suggesting that recent inbreeding could play a role in the disease. To better understand the impact of autozygosity on schizophrenia risk, we selected, from a cohort of 180 Italian patients, seven subjects with extremely high numbers of large ROHs that were likely due to recent inbreeding and characterized the mutational landscape within their ROHs using Whole Exome Sequencing and, gene set enrichment analysis. We identified a significant overlap (17%; empirical p-value = 0.0171) between genes inside ROHs affected by low frequency functional homozygous variants (107 genes) and the group of most promising candidate genes mutated in schizophrenia. Moreover, in four patients, we identified novel and extremely rare damaging mutations in the genes involved in neurodevelopment (*MEGF8*) and in GABA/glutamatergic synaptic transmission (*GAD1*, *FMN1*, *ANO2*). These results provide insights into the contribution of rare recessive mutations and inbreeding as risk factors for schizophrenia. ROHs that are likely due to recent inbreeding harbor a combination of predisposing low-frequency variants and extremely rare variants that have a high impact on pivotal biological pathways implicated in the disease. In addition, this study confirms that focusing on patients with high levels of homozygosity could be a useful prioritization strategy for discovering new high-impact mutations in genetically complex disorders.

## Background

Inbreeding is a well-established risk factor for recessive Mendelian diseases because it results in the presence of autozygosity regions, which are long runs of homozygosity (ROHs) in which the two alleles are identical by descent. These regions, indeed, increase the odds of bearing rare, recessive, deleterious mutations in a homozygous state (reviewed in [1]). Whether recent inbreeding contributes to the risk of complex diseases, such as schizophrenia, is less clear. A recent study showed a suggestive enrichment of long ROHs in schizophrenic patients, suggesting that large autozygosity regions due to recent inbreeding could play a role in the disease [2]. However, previous studies failed to detect a significant enrichment of rare recessive deleterious homozygous variants in the schizophrenic population [3, 4]. Mating between relatives accounts for fewer than 1% of marriages in industrialized countries [5]; therefore, the appearance of rare homozygous risk variants should be an ultra-rare event that is difficult to identify in the general population.

An established prioritization approach to identify these types of variants combines homozygosity mapping with whole exome sequencing (WES) [6]. Indeed, variant selection based on autozygosity regions has been used effectively to study the role of inbreeding and recessive genotypes, not only in Mendelian disorders but also in the pathogenesis of complex disorders, such as autism [7] and intellectual disability [8].

In the absence of pedigree information, a way to select patients with autozygosity regions is by selecting patients with a high portion of their genome contained within long ROHs. Patterns of homozygosity observed in different human populations suggest that large ROHs (several Mb long) result largely from recent inbreeding, whereas short ROHs are also common in outbred populations and derived mainly from population-level LD patterns [9, 10].

To better understand the impact of rare homozygous variants and of autozygosity on the risk of schizophrenia, we selected, from a cohort of 180 Italian patients, seven subjects with extremely high numbers of large ROHs and characterized the mutational landscape within their ROHs using WES and gene set enrichment analyses.

Our results provide insights into the contribution of rare recessive mutations and inbreeding as risk factors for schizophrenia.

## Materials and methods

The study was approved by the ethics committee of Lombardy Region (NP1581-01/14/2014). All participants provided written informed consent for DNA collection.

### Patients

The study involved the 180 patients previously subjected to CNV analysis using the Affymetrix Human Mapping GeneChip 6.0 arrays, as and reported by Magri and colleagues [11]. Recruitment and eligibility criteria are described in more details elsewhere [11]. Briefly, patients were enrolled, on a consecutive basis, from those voluntarily admitted to the Brescia University and Spedali Civili Psychiatric Unit between 1997 and 2008. Patients were of both sexes (63% males), with a mean age at recruitment of 39.1 years (sd = 12) (range = 17–76 years) and a mean age at onset of 26.4 (sd = 8.2) (range 15–57). They were Caucasian, of self-reported Italian descent for at least 2 generations and were unrelated to one another. All patients satisfied the DSM-IV-TR criteria for schizophrenia in the absence, during their lifespan, of co-morbidities with other DSM-IV-TR Axis I disorders, with the exception of nicotine and caffeine abuse. They had a level of understanding and attention sufficient to give true informed consent.

### Homozygosity analysis

ROHs were detected with the SVS7 software (Golden Helix) using genotype data already available [11]. ROHs larger than 4 Mb are indicative of autozygosity due to recent parental relatedness [9, 10]. Therefore, to select patients with ROHs likely due to recent inbreeding, we first selected those with at least one ROH larger than 4 Mb, and from those individuals, we selected those with the highest number of large ROHs (subjects falling in the upper quartile).

### Whole exome sequencing

WES was performed on gDNA using the AmpliSeq Exome kit (Life Technologies) and the Ion PI Chip on the Ion Proton System (Life Technologies). Sequence alignment to Hg19 and variant identification were performed with the Torrent Suite v.4.2.1 and Torrent Suite Variant Caller v.4.2.1 software. Because false-positive indels are a limitation of the Ion Torrent technology, the raw VCF files containing the list of genetic variants identified in each sample were filtered against an in-house developed list of false positive indels prior to annotation with ANNOVAR [12]. Middle range and small CNVs were determined from aligned reads using the germline CNV workflow in Ion Reporter 5.2. This tool is optimized for Ion Torrent data, with a specific baseline for AmpliSeq Exome experiments. Only CNVs identified with high confidence and consisting of < 100 kb were considered, because larger ones are better determined from genotyping array data. Raw sequencing data in the form of BAM files containing aligned reads are available through the SRA archive (PRJNA377832).

### Definition of candidate low frequency functional (LFF) variants in ROH regions

**Fig 1:**
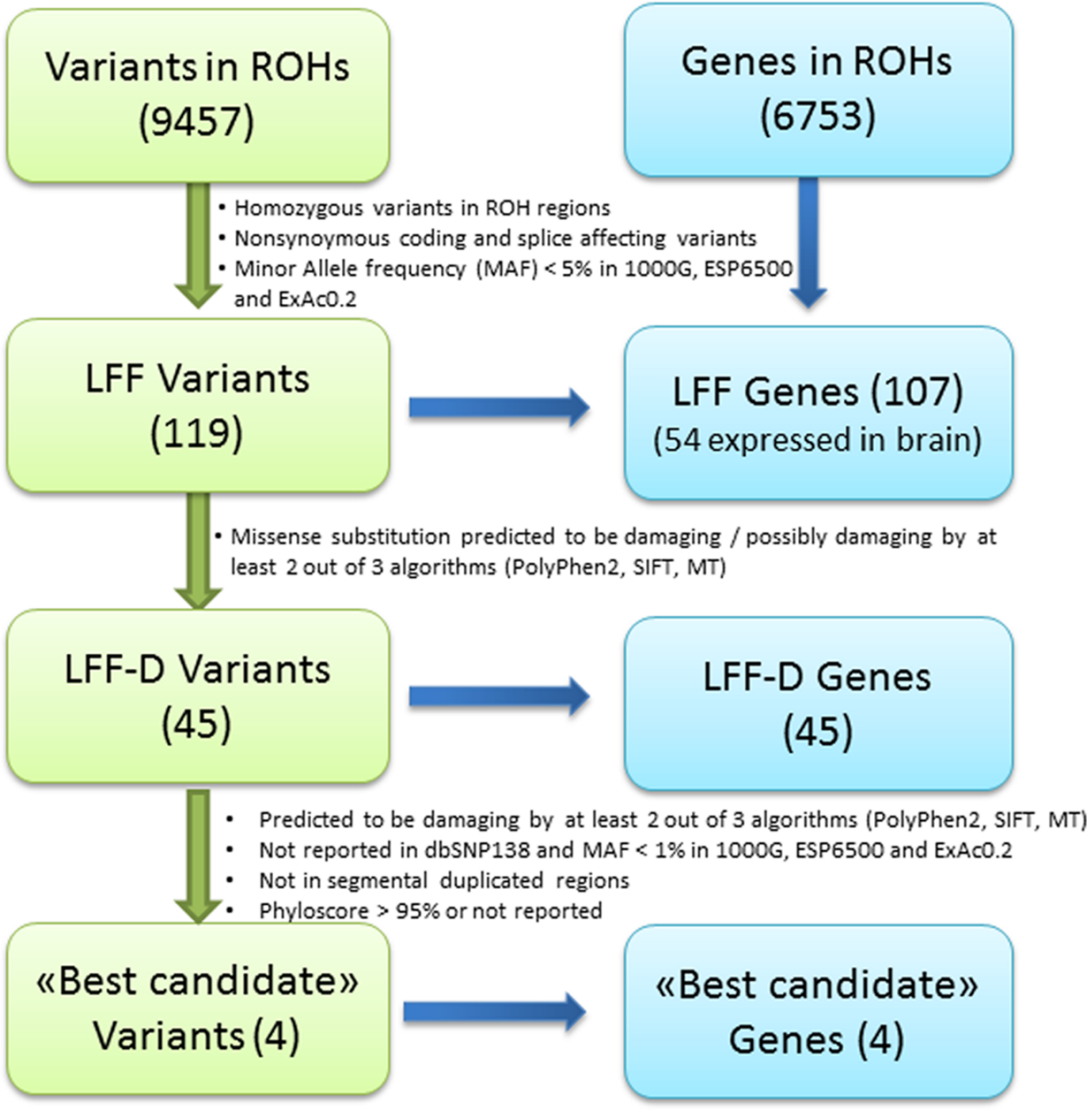
Pipeline for filtration of low frequency functional variants.

Following the filtration criteria reported in Fig 1, three lists of candidate variants were compiled: low frequency functional (LFF) variants, LFF damaging variants (LFF-D), and “best candidate” mutations. The “best candidate” mutations were confirmed by Sanger sequencing and genotyped in a cohort of 100 subjects (50 schizophrenic patients and 50 healthy controls) from the Italian population to exclude population specific polymorphisms.

### Gene set enrichment analysis

INRICH software [13] was used for gene set enrichment analyses of genes included in ROH regions over KEGG pathways, Gene Ontology (GO) categories and genes already reported to be associated with schizophrenia. The 1,826 genes included in the composite set of Purcell (SZ-composite set) [14] and the 348 genes included in the 108 loci found to be associated with schizophrenia by Ripke et al. (SZ-GWAS set) [15] were considered as genes associated with the disorder. Web GESTALT [16] was used to test for the enrichment of LFF and LFF-D genes in KEGG pathway and GO category gene sets. Finally, using random permutation, LFF and LFF-D gene lists were individually tested for enrichment over the SZ-composite set and the SZ-GWAS set.

Further details of the experimental procedures and data analyses are provided in S1 Appendix.

## Results

### Homozygosity analysis

Analysis of the SNP array data from the cohort of 180 Italian patients with schizophrenia revealed the presence of 27 subjects carrying, overall, 102 ROHs larger than 4Mb. Among them, seven patients fell in the upper quartile for the greatest number of long ROHs. These patients (ROH-individuals) had more than 4 long ROHs compared to a median value of 2 and more than 22 Mb of their genome were included in these ROHs compared to a median value of 8.7 Mb. ROH-individuals were further analyzed, extending the examination to all of their ROHs > 1 Mb. Table 1 summarizes the identified ROHs (see S1 Table for details). The clinical and demographic characteristics of the seven ROH-individuals are reported in the S1 Appendix.

**Table 1:** ROH regions in ROH-individuals.

Patient	Number of ROHs >1Mb	Median dimension (bp)	Max dimension (bp)	Amount of the genome within ROHs (bp)	Number of genes within ROHs
N° 1	26	2,468,705	13,889,443	82,551,340	764
N° 2	76	1,602,416	5,538,899	151,738,690	1,430
N° 3	28	2,610,206	12,005,057	108,730,336	861
N° 4	25	1,602,559	20,857,920	87,043,015	677
N° 5	51	2,890,666	12,377,762	219,863,310	1,538
N° 6	48	2,560,178	10,334,938	147,737,251	1,214
N° 7	42	3,268,145	9,268,459	144,180,390	1,216

Considering genes inside ROH regions of ROH-individuals, no significant enrichment was observed in any specific Gene Ontology category, KEGG pathway or list of genes previously reported to be associated with schizophrenia. S1 Fig shows the ROH regions and the regions reported to be associated with schizophrenia.

### Exome sequencing results

WES of ROH-individuals was performed with a mean coverage of 57–148X across the target region and with 77–94% of the bases covered by a sequencing depth of at least 20X. The analysis of the sequencing data led to the identification in each subject of 47,739–51,628 genetic variants with transition versus transversion ratios (Ti/Tv) of 2.49–2.60, comparable with those expected for human exomes (S2 Table). Considering only the ROH regions defined in S1 Table, each subject presented 991–1,988 variants, affecting 677–1,538 genes (Tables 1 and 2).

**Table 2:** Number of variants in ROH regions.

Patient	Variants	LFF variants ^a^	LFF variants in genes expressed in brain	LFF-D variants	“Best candidate” variants
N°1	1,064	15 (15)	6 (6)	7 (7)	
N°2	1,561	27 (25)	14 (14)	14 (14)	1
N°3	1,217	24 (16)	7 (7)	4 (4)	1
N°4	991	7 (7)	3 (3)	3 (3)	
N°5	1,988	24 (21)	13 (11)	9 (9)	
N°6	1,711	9 (9)	5 (5)	2 (2)	1
N°7	1,579	14 (14)	8 (8)	6 (6)	1
**All** ^**b**^	**9,457**	**119 (107)**	**56 (54)**	**45 (45)**	**4**

LFF = low frequency functional variants; LFF-D = low frequency damaging variants

^a^ in parenthesis the number of genes affected by the variants.

^b^ Total number of different variants

Since Patient 2 was carrier of the 3q29 deletion, the hemizygous region corresponding to this deletion was not included as a ROH, but was anyway analyzed for the potential presence of hemizygous damaging recessive mutations. No rare deleterious variants were observed.

### Candidate variants in ROH regions

Overall, 119 low frequency functional homozygous variants (LFF, MAF < 0.05) falling within 107 genes were identified inside the ROH regions, with 56 variants mapping to 54 genes expressed in the brain according to the EBI Expression Atlas database (FPKM > 1). WES-based analysis of small and intermediate CNVs excluded hemizygosity for these variants. CNV analysis revealed the presence of five small homozygous deletions inside ROHs, but all of them overlapped with segmental duplicated regions or highly polymorphic CNVs. For this reason, they were not considered for further analysis.

Among the LFF variants, 45 were predicted as damaging (LFF-D) (Fig 1). Table 2 reports the results for each ROH-individual, while the lists of candidate genes are reported in S3 Table. Four damaging variants, in four ROH-individuals, fulfilled the filtering criteria to be defined as “best candidate”. These variants were missense mutations mapping in the following genes: *ANO2* (Anoctamin 2), *FMN1* (Formin 1), *MEGF8* (Multiple EGF-Like-Domains 8) and *GAD1* (Glutamic acid decarboxylase 1). All of the “best candidate” variants were confirmed by Sanger sequencing. They were novel or extremely rare variants mapping in ROHs larger than 3 Mb and never reported in homozygosity in the three inspected variant databases (1000 Genome, Exome Variant server and ExAC 0.2) (Table 3). Moreover, none of them were detected in a cohort of 50 controls and 50 patients of Italian origin, confirming that they are neither common variants of the local population nor enriched in the schizophrenic group of patients from this population.

**Table 3:** List of “best candidate” variants.

Patient	Variants ^a^	Gene name	Gene ID	cDNA nucleotide change	AA change	SIFT (Pred.)	PP2_HDIV (Pred.)	MT (Pred.)	PP 100V	Frequency ^b^	ROH size ^c^
N°2	chr19:42840266G>A	*MEGF8*	NM_001271938	c.1012G>A	p.Ala338Thr	0.90 (T)	0.999 (D)	1 (D)	5.141	Novel	3.3 Mb
N°3	chr12:5963280G>A	*ANO2*	NM_001278596	c.562C>T	p.Arg188Trp	0.00 (D)	1.000 (D)	1 (D)	4.955	0.0000664	6.9 Mb
N°6	chr2:171687546A>G	*GAD1*	NM_000817	c.391A>G	p.Thr131Ala	0.02 (D)	0.986 (D)	1 (D)	8.962	Novel	5.8 Mb
N°7	chr15:33256378G>C	*FMN1*	NM_001103184	c.2399C>G	p.Ser800Cys	0.06 (T)	1.00 (D)	0.99 (D)	9.435	0.00000828	4.0 Mb

SIFT: Sorting Intolerant from Tolerant algorithm; PP2_HDIV: Polyphen2_HDIV; MT: Mutation Taster; D = predicted as probably damaging, deleterious or disease causing; T = tolerated.

^a^ positions are referred to the Hg19 assembly.

^b^ Frequency refers to allele frequency reported in the ExAC0.2 database; none of the variants have been reported in homozygous state.

^c^ Size of ROHs where the “best candidate” mutations were identified.

Applying the same “best candidate” criteria, we did not observe other homozygous variants in well-documented schizophrenia genes outside the ROH regions.

### Gene set enrichment analysis in LFF and LFF-D genes

The analyses of LFF and LFF-D genes in single ROH-individuals, as well as considering all seven ROH-individuals together, did not identify any significantly enriched KEGG or GO category containing at least two genes (Benjamini & Hochberg multiple test correction).

However, a significant enrichment of genes from the SZ-composite set was observed in the LFF genes (17%; empirical p-value = 0.0171). This enrichment was also suggestive among LFF-D genes (18%; empirical p-value = 0.0710). The LFF and LFF-D genes overlapping the SZ-composite set are listed in bold in S3 Table. No enrichment was found for genes mapping in the SZ-GWAS set.

## Discussion

Homozygosity mapping performed in 180 Italian patients with schizophrenia led to the identification of seven ROH-individuals whom we assumed were offspring of matings between relatives based on the high number of large ROHs in their genomes. Indeed, the genome pattern of homozygosity observed in different human populations suggests that large ROHs (several Mb long) are mostly due to recent inbreeding. Short ROHs, in contrast, are also common in outbred population and derived mainly from population-level Linkage Disequilibrium patterns [9, 10]. Characterization of the ROH mutational landscape of these patients revealed that genes affected by low frequency functional variants (LFF genes), despite not being enriched in specific GO categories or KEGG pathways, have a significant overlap with genes reported in the SZ-composite set of Purcell [14]. This set includes the most promising genes affected by rare mutations in schizophrenic patients (e.g., *de novo* schizophrenia nonsynonymous mutations, post synaptic density genes, calcium channel genes, and fragile X mental retardation protein gene targets). This enrichment remained suggestive even when only the predicted damaging mutations were taken into account. Since the enrichment was not the consequence of an increased density of these genes inside the ROH regions, this result suggests that at least some of the low frequency homozygous functional mutations identified are involved with the clinical phenotype of the patients. Our results, therefore, point to the presence of rare homozygous schizophrenic risk variants in subjects bearing long ROHs that are likely due to recent inbreeding. That long ROHs regions could be enriched for homozygous schizophrenic risk variants is also suggested by a recent paper by Johnson and colleagues, who failed to identify a reliable association between ROH burden and schizophrenia but found p-values that approached significant associations when large ROHs were considered separately [2]. Johnson’s results and ours indirectly support the hypothesis reported by Szpiech and collaborators [17], whereby long ROHs are more enriched for deleterious homozygous variants because they are likely generated by a recent inbreeding event and natural selection has not had time to purify them from deleterious alleles. Considering that subjects harboring long ROHs due to recent inbreeding represent only a small fraction of schizophrenic patients, rare recessive deleterious variants would be difficult to observe in a schizophrenic population without selecting for patients who have long ROHs. This would probably explain why an increased burden of rare recessive deleterious variants was not observed in previous studies [3, 4].

Application of strict filtration criteria to low frequency functional variants (LFF) led to the identification of four “best candidate” mutations, mapping in the *MEGF8*, *FMN1*, *ANO2* and *GAD1* genes.

The *MEGF8* gene encodes for a single-pass type I membrane protein containing multiple EGF-like domains and is reported to be involved in several developmental processes, including axon guidance [18]. Homozygous recessive *MEGF8* mutations have been described in five patients with the Carpenter syndrome subtype associated with defective lateralization [19]. Mild to moderate intellectual disability have been observed in some Carpenter subjects (OMIM:201000) although these symptoms have not been reported in the five cases with the subtype due to *MEGF8* mutations. A patient with a molecularly uncharacterized type of Carpenter syndrome who was also suffering from schizophrenia was reported [20]. A *MEGF8* mutation was found in the patient with the 3q29 deletion (patient 2), a CNV highly penetrant in schizophrenia, but also associated with a range of other neurodevelopmental phenotypes [21]. Thus, it is possible that, in our patient, the *MEGF8* point mutation might contribute to the general neurodevelopmental disease risk primarily conferred by the 3q29 deletion or might more specifically modulate the schizophrenic phenotype.

**Fig 2:**
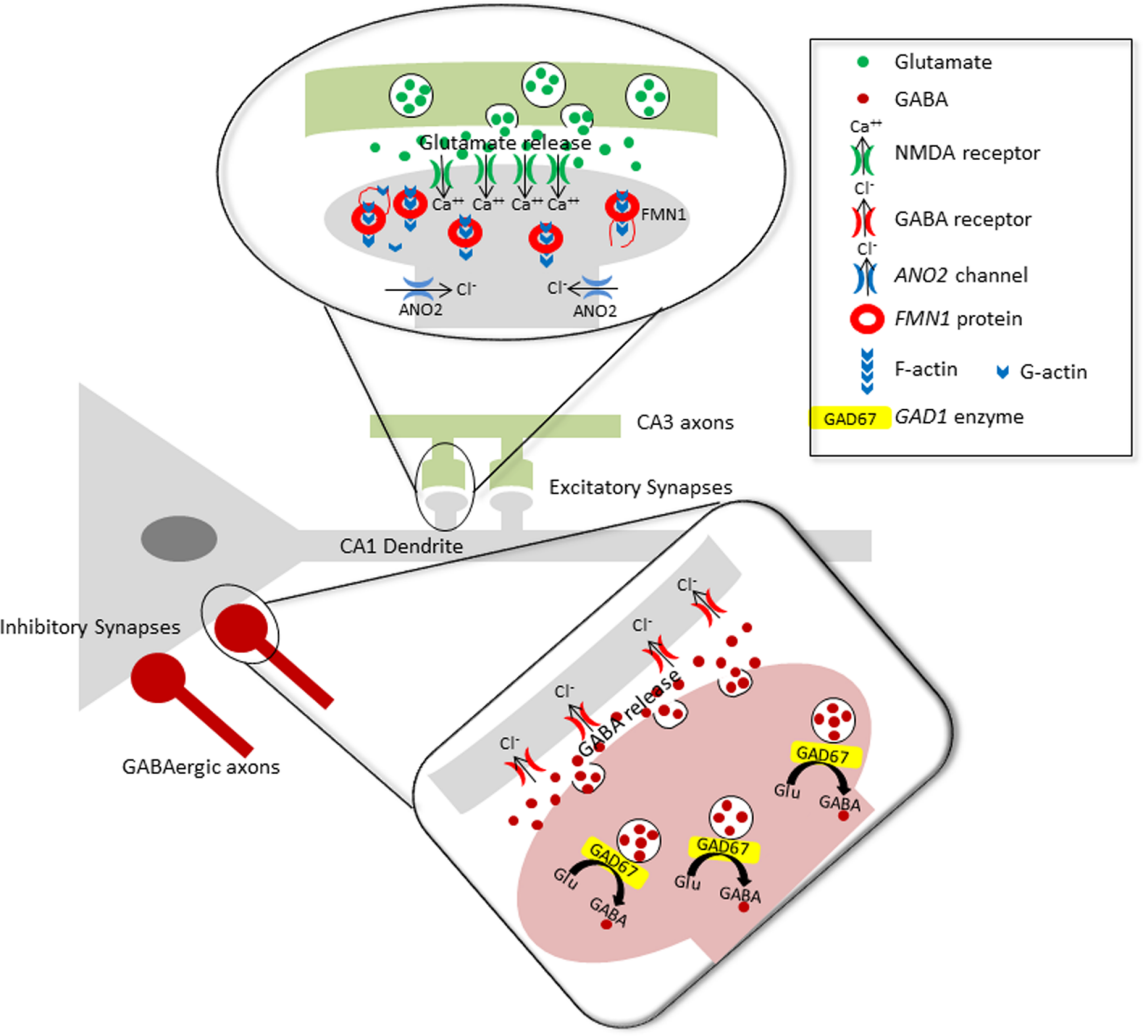
Localization at the synaptic level of the *FMN1*, *ANO2* and *GAD1* gene products on a hippocampal neuronal circuit. FMN1 protein and calcium-activated ANO2 chloride channels are expressed in the dendritic spines of excitatory synapses [22, 23], whereas GAD67 (the enzyme produced by the *GAD1* gene) is expressed in the axonal termini of inhibitory GABAergic interneurons. It has been shown that impairment of these genes induces: dendritic arborization abnormalities (*FMN1*) [23], alterations in action potential duration and in the threshold for action potential generation (*ANO2*) [22], and the dysfunctional synthesis of the GABA neurotransmitter (*GAD1*) [24]. At different levels, the impairment of these genes might affect the complex excitatory/inhibitory balance across cortical circuits that is believed to be altered in schizophrenic patients [25].

The other three “best candidate” mutations identified affect genes involved in synaptic transmission, in particular the GABA/glutamatergic pathways (Fig 2).

The *FMN1* gene encodes for formin1, a protein involved in the formation of adherent junctions and the polymerization of linear actin cables; it also plays a role in dendritogenesis and synaptogenesis in mouse hippocampal neurons [23], where its expression levels are related to the number of primary dendrites and glutamatergic synaptic inputs. A microdeletion encompassing part of the *FMN1* gene has been recently reported in a patient with early-onset obsessive-compulsive disorder [26]; interestingly, this deletion includes the *FMN1* exon (exon 5) containing the described mutation.

The *ANO2* gene belongs to a family of calcium-activated chloride channels (CaCCs), and it is mainly expressed in olfactory sensory neurons [27] and photoreceptor termini [28]. This gene is also expressed in hippocampal pyramidal neurons, where it encodes for CaCCs that reside in the vicinity of voltage-gated Ca^2+^ channels to regulate spike duration and proximity to NMDA receptors to modulate excitatory synaptic responses [22].

Finally, *GAD1* is one of the two genes encoding for the enzymes that catalyze the production of gamma-aminobutyric acid (GABA) from L-glutamic acid. Of the “best candidate” genes, this is the most intriguing, because reduced *GAD1* mRNA and protein levels have been consistently observed in *post-mortem* brains of schizophrenia patients [29, 30]. Common polymorphisms in the proximal *GAD1* promoter have also been suggested to act as genetic risk factors for schizophrenia [31–33]. The *GAD1* missense mutation identified here, leading to the substitution of a highly conserved amino acid residue, might result in reduced enzymatic activity, mimicking the effect of a down-regulation of protein expression. The identification of novel and extremely rare damaging mutations in genes belonging to biological pathways strongly implicated in the pathogenesis of schizophrenia suggests the involvement of rare recessive deleterious genotypes in the etiopathogenesis of the disease. However, we are aware that the actual impact of our “best candidate” homozygous variants on schizophrenic phenotype can only be inferred from large association studies.

The detection, in patients with high levels of homozygosity, of an enrichment of low frequency functional variants in genes previously reported associated with schizophrenia and the identification of four novel or extremely rare risk variants affecting pivotal pathways involved in schizophrenia suggest that the clinical phenotype of these patients could be the result of the interplay between strong risk variants and predisposing genetic backgrounds. This, once again, supports the results of Szpiech and collaborators reporting that recent inbreeding magnifies the occurrence of extremely rare strongly deleterious mutations, as well as of mild deleterious variants [17].

Despite the detailed genetic characterization of ROH regions, some limitations could have affected our analysis. Based on data in the literature [9, 10], we had assumed that the high number of large ROHs detected in the seven schizophrenic patients was the consequence of recent inbreeding. However, we are aware that recent parental relatedness can be firmly inferred only from pedigree information, which was not available for these patients.

Moreover, in these patients, we identified some novel/rare mutations and identified them as risk factors for schizophrenia because they are deleterious and/or affect genes previously implicated in the disease. The deleteriousness of these variants, however, is based only on bioinformatics predictions. Future functional studies in cellular models are needed to confirm the biological role of these variants.

Finally, this study was limited to the coding portion of the genome; thus the spectrum of genetic variability relevant for schizophrenia was not fully addressed. Indeed, several loci associated with the disorder in GWAS mapped to intergenic regions, suggesting an important role of regulatory variants. An analysis of noncoding variants is therefore needed to fully understand the role played by rare recessive variants in schizophrenia.

## Conclusions

Despite the limitations reported, our results provide insights into the contribution of rare recessive mutations and inbreeding as risk factors for schizophrenia. They suggest that ROHs that are likely due to recent inbreeding harbor a combination of predisposing low frequency variants and extremely rare variants with high impact in pivotal biological pathways implicated in the disease. In addition, this study confirms that focusing on patients with high levels of homozygosity could be a useful prioritization strategy to discover new high-impact mutations in genetically complex disorders.

## Supporting information

S1 Table ROH regions identified in ROH-individuals. (PDF)

S2 Table Exome sequencing results. (PDF)

S3 Table LFF and LFF-D gene lists. (PDF)

S1 Fig ROH distribution across the analyzed subjects. The circular representation shows the distribution of ROHs (black blocks) in the seven ROH-individuals, which are reported in ascending order from the external circle as white/gray tracks. Green tracks show the genomic localization of genes/genomic regions already associated with schizophrenia in previous studies. From the external circle: SZ-GWAS set, SZ-composite set, genes from the SZGR database. Black marks on chromosome ideograms represent loci shared between schizophrenia genes/regions and our ROHs.
(PDF)

S1 Appendix Supplementary methods and clinical and demographic descriptions of ROH-individuals. (PDF)
