# Supplementary material for: Exome sequencing in schizophrenic patients with high levels of homozygosity identifies novel and extremely rare mutations in the GABA/glutamatergic pathways
Source: PLoS One. 2017 Aug 7;12(8):e0182778. doi: 10.1371/journal.pone.0182778 (PMC5546675; doi:10.1371/journal.pone.0182778)
Supplement: S1 Table — (PDF) [file pone.0182778.s001.pdf]

S1 Table. ROH regions identified in ROH-individuals

Sample	Chromosome	Start position	End position	Length (bp)	N. SNPs	N. Missings[a]	N. Heterozygotes[b]
**Patient N° 1**	1	237 774 614	241 183 190	3 408 576	602	2	1
**Patient N° 1**	2	32 190 504	36 112 278	3 921 774	579	5	1
**Patient N° 1**	2	36 135 389	38 512 063	2 376 674	503	3	1
**Patient N° 1**	2	38 542 573	43 190 388	4 647 815	677	4	1
**Patient N° 1**	2	188 684 507	189 855 674	1 171 167	77	0	1
**Patient N° 1**	3	57 074 218	58 232 659	1 158 441	107	1	1
**Patient N° 1**	3	185 048 314	188 870 822	3 822 508	634	5	1
**Patient N° 1**	3	188 878 969	190 286 886	1 407 917	262	2	1
**Patient N° 1**	6	46 867 717	49 751 612	2 883 895	285	2	1
**Patient N° 1**	6	49 778 621	52 612 904	2 834 283	316	3	1
**Patient N° 1**	6	65 906 330	66 904 407	998 077	103	0	1
**Patient N° 1**	7	68 891 147	70 046 675	1 155 528	69	0	1
**Patient N° 1**	7	138 764 060	152 653 503	13 889 443	1481	5	1
**Patient N° 1**	10	32 537 928	33 563 893	1 025 965	116	0	1
**Patient N° 1**	10	102 884 730	103 922 896	1 038 166	65	0	1
**Patient N° 1**	11	206 767	4 099 051	3 892 284	414	2	1
**Patient N° 1**	11	133 051 091	134 893 915	1 842 824	425	5	0
**Patient N° 1**	12	25 138 016	27 698 751	2 560 735	407	5	1
**Patient N° 1**	12	60 093 791	61 383 227	1 289 436	75	0	1
**Patient N° 1**	13	70 997 511	72 765 304	1 767 793	229	2	1
**Patient N° 1**	13	72 771 714	75 810 761	3 039 047	476	1	1
**Patient N° 1**	13	82 315 799	90 151 254	7 835 455	642	4	1
**Patient N° 1**	16	59 512 010	60 996 700	1 484 690	180	1	1
**Patient N° 1**	18	48 194 641	50 150 118	1 955 477	237	1	1
**Patient N° 1**	22	39 756 854	46 459 132	6 702 278	787	5	1
**Patient N° 1**	22	46 480 602	50 921 694	4 441 092	892	5	1
**Patient N° 2**	1	67 526 029	70 225 340	2 699 311	317	5	1
**Patient N° 2**	1	75 280 496	76 627 868	1 347 372	134	2	1
**Patient N° 2**	1	76 975 717	80 150 181	3 174 464	328	5	1
**Patient N° 2**	1	234 801 409	237 087 952	2 286 543	291	5	1
**Patient N° 2**	2	1 318 413	2 608 466	1 290 053	188	5	1

**Patient N° 2**	2	4 034 446	5 258 167	1 223 721	205	5	1
**Patient N° 2**	2	34 538 100	36 420 568	1 882 468	249	5	1
**Patient N° 2**	2	37 023 044	38 693 158	1 670 114	311	5	1
**Patient N° 2**	2	38 847 132	40 332 851	1 485 719	134	5	1
**Patient N° 2**	2	40 539 251	42 258 948	1 719 697	299	5	1
**Patient N° 2**	2	42 267 462	43 574 242	1 306 780	204	5	1
**Patient N° 2**	2	45 061 926	46 456 582	1 394 656	380	5	1
**Patient N° 2**	2	188 072 104	189 118 320	1 046 216	72	0	1
**Patient N° 2**	2	206 464 465	207 948 857	1 484 392	221	3	1
**Patient N° 2**	3	46 656 351	50 158 191	3 501 840	80	3	1
**Patient N° 2**	3	114 206 587	115 239 407	1 032 820	75	1	1
**Patient N° 2**	3	137 662 094	139 974 934	2 312 840	214	5	1
**Patient N° 2**	4	4 391 648	5 975 174	1 583 526	387	5	1
**Patient N° 2**	4	32 606 917	35 911 736	3 304 819	186	5	0
**Patient N° 2**	4	35 931 138	37 518 563	1 587 425	232	5	1
**Patient N° 2**	4	39 410 182	41 222 268	1 812 086	267	5	1
**Patient N° 2**	4	41 769 950	47 308 849	5 538 899	457	5	0
**Patient N° 2**	4	53 067 343	55 201 854	2 134 511	205	5	0
**Patient N° 2**	4	55 921 535	59 214 630	3 293 095	422	5	1
**Patient N° 2**	4	59 907 023	64 381 142	4 474 119	390	5	0
**Patient N° 2**	4	65 436 643	68 412 148	2 975 505	302	5	1
**Patient N° 2**	4	68 976 217	73 530 159	4 553 942	298	5	1
**Patient N° 2**	4	73 575 684	75 279 982	1 704 298	112	5	1
**Patient N° 2**	4	100 575 711	101 942 847	1 367 136	142	4	1
**Patient N° 2**	4	134 026 066	135 775 238	1 749 172	109	1	1
**Patient N° 2**	4	166 337 135	169 017 774	2 680 639	273	2	1
**Patient N° 2**	4	169 686 795	172 560 014	2 873 219	233	1	1
**Patient N° 2**	4	172 949 886	175 376 524	2 426 638	227	4	1
**Patient N° 2**	4	175 621 502	179 527 417	3 905 915	498	5	1
**Patient N° 2**	4	179 536 770	181 266 629	1 729 859	261	5	1
**Patient N° 2**	4	181 289 859	182 397 985	1 108 126	269	5	1
**Patient N° 2**	5	98 726 889	99 982 292	1 255 403	79	2	1
**Patient N° 2**	6	27 312 078	28 502 550	1 190 472	68	1	1

**Patient N° 2**	6	120 386 153	121 862 617	1 476 464	105	3	1
**Patient N° 2**	6	131 845 152	133 610 298	1 765 146	237	5	1
**Patient N° 2**	6	133 710 214	137 882 681	4 172 467	453	5	1
**Patient N° 2**	6	140 024 345	144 535 974	4 511 629	425	5	1
**Patient N° 2**	6	145 635 385	148 671 794	3 036 409	328	5	1
**Patient N° 2**	6	149 293 235	150 476 086	1 182 851	131	5	1
**Patient N° 2**	6	164 049 551	165 798 963	1 749 412	325	5	0
**Patient N° 2**	7	55 724 445	56 819 848	1 095 403	66	1	1
**Patient N° 2**	7	89 687 264	90 695 079	1 007 815	103	1	1
**Patient N° 2**	7	111 782 891	113 040 467	1 257 576	135	3	1
**Patient N° 2**	7	123 855 871	124 935 779	1 079 908	89	0	1
**Patient N° 2**	7	145 422 195	146 577 200	1 155 005	96	0	1
**Patient N° 2**	7	151 548 007	152 726 381	1 178 374	119	4	1
**Patient N° 2**	7	152 801 121	154 246 735	1 445 614	266	5	1
**Patient N° 2**	8	51 654 055	52 929 150	1 275 095	87	2	1
**Patient N° 2**	8	67 194 171	68 568 185	1 374 014	69	1	1
**Patient N° 2**	10	57 720 276	58 803 817	1 083 541	81	1	1
**Patient N° 2**	10	124 586 574	125 942 586	1 356 012	242	5	1
**Patient N° 2**	10	125 954 944	128 424 324	2 469 380	431	5	1
**Patient N° 2**	10	128 581 683	129 631 391	1 049 708	219	5	0
**Patient N° 2**	10	129 651 938	130 769 970	1 118 032	262	5	1
**Patient N° 2**	10	134 313 336	135 434 551	1 121 215	88	5	1
**Patient N° 2**	11	88 286 929	89 904 336	1 617 407	77	3	1
**Patient N° 2**	12	52 799 301	54 316 307	1 517 006	183	5	1
**Patient N° 2**	12	55 214 165	57 163 316	1 949 151	124	5	1
**Patient N° 2**	12	57 208 230	61 001 641	3 793 411	314	5	1
**Patient N° 2**	12	61 019 462	62 455 504	1 436 042	122	5	0
**Patient N° 2**	13	89 275 195	90 506 508	1 231 313	84	1	1
**Patient N° 2**	15	23 046 404	24 583 066	1 536 662	155	4	1
**Patient N° 2**	15	25 033 395	26 181 335	1 147 940	213	5	0
**Patient N° 2**	15	26 278 561	27 343 875	1 065 314	187	5	1
**Patient N° 2**	15	27 412 578	29 426 782	2 014 204	153	5	1
**Patient N° 2**	15	63 469 846	66 428 824	2 958 978	213	5	1

**Patient N° 2**	15	66 929 574	68 611 489	1 681 915	213	5	1
**Patient N° 2**	15	68 686 077	70 967 554	2 281 477	274	5	1
**Patient N° 2**	19	37 048 117	39 088 461	2 040 344	112	5	1
**Patient N° 2**	19	39 152 908	40 251 805	1 098 897	107	5	0
**Patient N° 2**	19	40 347 035	43 646 784	3 299 749	193	5	1
**Patient N° 3**	1	7 245 434	12 915 847	5 670 413	600	5	0
**Patient N° 3**	1	13 821 129	15 188 019	1 366 890	281	1	1
**Patient N° 3**	2	41 179 801	45 380 085	4 200 284	688	5	1
**Patient N° 3**	2	203 532 304	204 825 286	1 292 982	76	0	1
**Patient N° 3**	4	32 301 265	33 642 614	1 341 349	69	0	1
**Patient N° 3**	5	175 078 991	176 130 808	1 051 817	105	0	1
**Patient N° 3**	6	28 077 374	29 353 408	1 276 034	89	0	1
**Patient N° 3**	6	108 856 449	110 690 440	1 833 991	163	0	1
**Patient N° 3**	6	121 884 929	126 215 101	4 330 172	501	2	1
**Patient N° 3**	6	127 414 801	134 625 104	7 210 303	881	5	0
**Patient N° 3**	6	134 630 783	138 259 865	3 629 082	390	5	1
**Patient N° 3**	8	60 540 056	61 818 964	1 278 908	107	0	1
**Patient N° 3**	8	68 541 850	72 864 240	4 322 390	565	2	1
**Patient N° 3**	9	15 973 712	23 078 402	7 104 690	1343	5	1
**Patient N° 3**	9	75 519 251	80 211 487	4 692 236	722	3	1
**Patient N° 3**	10	57 691 482	58 809 636	1 118 154	84	0	1
**Patient N° 3**	10	75 778 687	77 170 685	1 391 998	67	0	1
**Patient N° 3**	10	95 859 929	97 069 762	1 209 833	96	0	1
**Patient N° 3**	10	130 248 440	133 996 225	3 747 785	841	5	0
**Patient N° 3**	11	8 380 719	10 848 430	2 467 711	309	2	1
**Patient N° 3**	11	11 670 871	23 675 928	12 005 057	1868	5	1
**Patient N° 3**	11	100 146 092	102 898 793	2 752 701	381	0	1
**Patient N° 3**	12	1 024 688	7 945 384	6 920 696	1160	4	1
**Patient N° 3**	12	73 153 191	74 509 618	1 356 427	98	1	1
**Patient N° 3**	12	111 288 843	113 257 970	1 969 127	88	0	1
**Patient N° 3**	18	22 549 451	33 314 352	10 764 901	1124	2	1
**Patient N° 3**	18	33 323 857	43 982 231	10 658 374	1146	5	1
**Patient N° 3**	19	15 113 675	16 879 706	1 766 031	193	0	1

**Patient N° 4**	1	42 354 233	43 504 358	1 150 125	115	1	1
**Patient N° 4**	2	112 421 566	113 940 839	1 519 273	162	1	1
**Patient N° 4**	2	127 880 106	129 016 127	1 136 021	108	1	1
**Patient N° 4**	2	155 330 994	156 933 553	1 602 559	139	1	1
**Patient N° 4**	3	139 253 871	149 833 716	10 579 845	1260	2	1
**Patient N° 4**	4	22 699 639	30 087 239	7 387 600	972	3	1
**Patient N° 4**	4	127 470 966	129 223 362	1 752 396	111	0	1
**Patient N° 4**	4	143 472 696	145 031 841	1 559 145	78	0	1
**Patient N° 4**	5	23 713 221	24 837 899	1 124 678	78	0	1
**Patient N° 4**	5	42 398 305	44 279 163	1 880 858	91	1	1
**Patient N° 4**	5	61 194 553	62 504 074	1 309 521	112	0	1
**Patient N° 4**	8	111 455 605	113 617 156	2 161 551	107	0	1
**Patient N° 4**	9	123 079 481	124 368 674	1 289 193	87	0	1
**Patient N° 4**	10	25 821 161	29 173 102	3 351 941	415	1	1
**Patient N° 4**	10	114 907 055	122 749 548	7 842 493	1086	3	1
**Patient N° 4**	11	3 129 965	6 743 665	3 613 700	620	4	1
**Patient N° 4**	12	28 268 837	29 666 890	1 398 053	148	1	1
**Patient N° 4**	13	19 552 050	20 654 610	1 102 560	85	0	1
**Patient N° 4**	14	31 227 836	33 272 983	2 045 147	270	1	1
**Patient N° 4**	14	40 277 393	41 699 887	1 422 494	152	0	1
**Patient N° 4**	15	40 877 322	42 223 259	1 345 937	75	0	1
**Patient N° 4**	15	72 030 941	74 022 114	1 991 173	82	0	1
**Patient N° 4**	18	40 196 128	61 054 048	20 857 920	2774	5	1
**Patient N° 4**	18	71 623 644	78 015 057	6 391 413	1080	1	1
**Patient N° 4**	22	31 436 822	32 664 241	1 227 419	91	0	1
**Patient N° 5**	1	83 057 803	91 556 657	8 498 854	1019	5	1
**Patient N° 5**	1	91 566 457	94 585 009	3 018 552	274	2	1
**Patient N° 5**	1	95 056 901	103 110 933	8 054 032	859	5	0
**Patient N° 5**	1	103 128 845	108 035 245	4 906 400	367	5	0
**Patient N° 5**	1	108 056 892	118 223 984	10 167 092	1322	5	0
**Patient N° 5**	1	118 225 681	120 520 715	2 295 034	227	3	0
**Patient N° 5**	1	175 831 284	176 935 671	1 104 387	79	1	1
**Patient N° 5**	1	182 696 117	183 889 552	1 193 435	82	2	1

**Patient N° 5**	2	104 047 330	105 176 835	1 129 505	86	0	1
**Patient N° 5**	2	115 683 514	124 018 896	8 335 382	875	2	1
**Patient N° 5**	2	124 869 037	133 165 560	8 296 523	745	5	1
**Patient N° 5**	2	133 778 855	137 556 677	3 777 822	463	5	0
**Patient N° 5**	2	137 565 841	138 787 927	1 222 086	156	5	1
**Patient N° 5**	2	141 848 910	150 177 878	8 328 968	709	5	0
**Patient N° 5**	2	210 386 175	212 274 937	1 888 762	171	2	1
**Patient N° 5**	4	97 549 082	99 023 151	1 474 069	69	2	1
**Patient N° 5**	5	88 888 645	91 478 027	2 589 382	220	3	1
**Patient N° 5**	5	91 556 374	99 664 527	8 108 153	700	4	1
**Patient N° 5**	5	99 699 931	103 200 636	3 500 705	251	3	1
**Patient N° 5**	5	121 426 122	122 755 334	1 329 212	160	1	1
**Patient N° 5**	5	122 762 229	133 075 880	10 313 651	1101	5	1
**Patient N° 5**	5	133 147 901	137 618 925	4 471 024	538	5	1
**Patient N° 5**	5	137 759 010	144 874 969	7 115 959	696	4	1
**Patient N° 5**	5	144 898 571	146 442 247	1 543 676	159	3	1
**Patient N° 5**	5	146 727 485	155 014 075	8 286 590	1120	5	1
**Patient N° 5**	5	155 027 573	160 448 976	5 421 403	768	4	1
**Patient N° 5**	5	160 455 580	162 960 118	2 504 538	259	5	0
**Patient N° 5**	5	165 156 306	171 626 110	6 469 804	1182	5	1
**Patient N° 5**	5	172 230 862	175 007 610	2 776 748	616	3	1
**Patient N° 5**	6	28 254 988	29 356 046	1 101 058	76	0	1
**Patient N° 5**	6	78 549 277	84 234 144	5 684 867	555	5	1
**Patient N° 5**	6	84 277 037	91 859 454	7 582 417	823	5	1
**Patient N° 5**	6	91 944 598	103 652 391	11 707 793	1133	5	1
**Patient N° 5**	6	104 628 972	109 558 471	4 929 499	588	2	1
**Patient N° 5**	6	110 813 928	123 191 690	12 377 762	1158	5	1
**Patient N° 5**	6	123 196 340	125 470 012	2 273 672	308	1	1
**Patient N° 5**	6	168 508 297	170 905 977	2 397 680	402	2	0
**Patient N° 5**	7	26 512 076	28 499 363	1 987 287	304	5	1
**Patient N° 5**	7	28 507 533	31 002 540	2 495 007	454	5	1
**Patient N° 5**	7	125 484 555	126 571 077	1 086 522	109	2	1
**Patient N° 5**	8	78 407 296	79 753 179	1 345 883	81	1	1

**Patient N° 5**	10	57 691 482	59 158 985	1 467 503	99	0	1
**Patient N° 5**	11	46 297 631	49 188 297	2 890 666	68	2	1
**Patient N° 5**	13	22 559 190	24 835 739	2 276 549	507	2	1
**Patient N° 5**	13	25 573 633	31 767 035	6 193 402	1083	5	1
**Patient N° 5**	13	55 281 772	57 403 877	2 122 105	78	1	1
**Patient N° 5**	14	27 531 070	31 288 146	3 757 076	355	2	1
**Patient N° 5**	14	67 995 628	69 045 876	1 050 248	111	0	1
**Patient N° 5**	16	70 758 855	72 979 063	2 220 208	167	1	1
**Patient N° 5**	18	69 037 838	70 567 020	1 529 182	273	3	1
**Patient N° 5**	18	70 568 619	73 833 795	3 265 176	584	5	1
**Patient N° 6**	1	224 932 570	225 988 818	1 056 248	86	0	1
**Patient N° 6**	2	40 257 960	42 350 487	2 092 527	405	3	1
**Patient N° 6**	2	153 273 219	158 071 800	4 798 581	512	5	1
**Patient N° 6**	2	158 094 105	160 321 840	2 227 735	242	5	1
**Patient N° 6**	2	160 861 218	166 965 458	6 104 240	499	5	0
**Patient N° 6**	2	166 988 362	169 154 462	2 166 100	220	4	1
**Patient N° 6**	2	171 265 546	177 067 780	5 802 234	693	5	1
**Patient N° 6**	3	170 935 941	172 368 980	1 433 039	251	5	1
**Patient N° 6**	3	172 372 521	174 926 485	2 553 964	307	5	1
**Patient N° 6**	4	46 238 737	49 063 479	2 824 742	213	1	0
**Patient N° 6**	4	53 830 757	55 124 460	1 293 703	161	0	1
**Patient N° 6**	4	150 933 554	157 163 395	6 229 841	645	5	0
**Patient N° 6**	4	157 165 831	158 533 769	1 367 938	144	3	1
**Patient N° 6**	5	101 216 027	102 986 653	1 770 626	117	1	1
**Patient N° 6**	6	27 296 775	28 641 689	1 344 914	83	2	1
**Patient N° 6**	8	51 462 216	52 496 221	1 034 005	71	2	1
**Patient N° 6**	8	87 791 299	95 244 047	7 452 748	603	5	0
**Patient N° 6**	8	95 497 989	100 997 714	5 499 725	601	5	0
**Patient N° 6**	8	101 117 734	103 112 417	1 994 683	283	5	0
**Patient N° 6**	8	104 363 338	114 698 276	10 334 938	850	5	1
**Patient N° 6**	8	114 724 656	117 817 256	3 092 600	265	5	1
**Patient N° 6**	8	117 917 260	123 325 109	5 407 849	626	5	0
**Patient N° 6**	8	123 980 448	127 613 934	3 633 486	516	5	1

**Patient N° 6**	8	128 440 928	130 353 593	1 912 665	324	5	1
**Patient N° 6**	8	130 864 813	135 537 174	4 672 361	893	5	1
**Patient N° 6**	8	135 541 720	136 767 239	1 225 519	198	3	1
**Patient N° 6**	8	136 772 337	139 157 175	2 384 838	331	4	1
**Patient N° 6**	9	86 203 042	87 409 367	1 206 325	150	2	1
**Patient N° 6**	11	339 969	2 766 234	2 426 265	236	5	0
**Patient N° 6**	11	2 774 839	3 838 910	1 064 071	128	0	1
**Patient N° 6**	11	3 865 786	6 724 381	2 858 595	526	5	1
**Patient N° 6**	11	6 729 493	9 295 884	2 566 391	447	5	1
**Patient N° 6**	11	9 352 951	11 422 750	2 069 799	406	5	1
**Patient N° 6**	11	84 838 195	85 869 944	1 031 749	88	2	1
**Patient N° 6**	11	105 483 661	106 519 130	1 035 469	104	0	1
**Patient N° 6**	12	85 578 064	89 252 667	3 674 603	158	1	1
**Patient N° 6**	14	40 183 285	41 265 598	1 082 313	117	0	1
**Patient N° 6**	14	76 562 250	83 326 226	6 763 976	976	5	1
**Patient N° 6**	15	74 338 939	79 151 286	4 812 347	328	5	1
**Patient N° 6**	15	79 321 517	82 112 627	2 791 110	465	5	1
**Patient N° 6**	15	83 670 073	86 320 952	2 650 879	270	1	1
**Patient N° 6**	15	86 332 964	88 120 669	1 787 705	390	5	0
**Patient N° 6**	15	88 390 517	91 622 281	3 231 764	420	5	1
**Patient N° 6**	15	95 160 747	98 687 947	3 527 200	691	5	1
**Patient N° 6**	15	98 704 009	101 679 433	2 975 424	587	5	1
**Patient N° 6**	16	85 533 240	87 100 306	1 567 066	514	5	1
**Patient N° 6**	16	87 502 815	90 163 275	2 660 460	292	5	1
**Patient N° 6**	21	14 597 009	18 838 900	4 241 891	496	2	1
**Patient N° 7**	2	23 871 024	25 359 254	1 488 230	89	0	1
**Patient N° 7**	2	152 006 025	158 447 571	6 441 546	634	5	1
**Patient N° 7**	2	159 788 918	164 300 859	4 511 941	339	5	1
**Patient N° 7**	2	164 619 926	168 786 418	4 166 492	410	5	1
**Patient N° 7**	3	99 051 310	100 550 136	1 498 826	105	1	1
**Patient N° 7**	4	177 572 077	179 374 122	1 802 045	218	5	1
**Patient N° 7**	5	59 825 300	60 904 429	1 079 129	73	1	1
**Patient N° 7**	6	27 296 775	28 769 671	1 472 896	88	1	1

**Patient N° 7**	7	4 384 426	7 734 829	3 350 403	393	4	1
**Patient N° 7**	8	109 774 461	110 758 319	983 858	75	0	1
**Patient N° 7**	10	104 217 415	105 313 230	1 095 815	67	1	1
**Patient N° 7**	11	19 130 993	23 525 279	4 394 286	802	5	1
**Patient N° 7**	11	24 159 696	28 607 245	4 447 549	483	5	1
**Patient N° 7**	11	103 950 742	105 937 044	1 986 302	124	2	1
**Patient N° 7**	11	132 831 793	134 631 823	1 800 030	415	5	0
**Patient N° 7**	13	52 374 489	53 501 200	1 126 711	90	0	1
**Patient N° 7**	13	72 278 579	75 389 543	3 110 964	514	2	1
**Patient N° 7**	13	76 739 402	80 285 558	3 546 156	424	5	1
**Patient N° 7**	13	80 289 116	85 097 830	4 808 714	469	5	1
**Patient N° 7**	13	85 715 146	92 371 090	6 655 944	618	5	1
**Patient N° 7**	14	62 665 000	67 667 402	5 002 402	468	5	1
**Patient N° 7**	14	69 011 205	75 867 306	6 856 101	915	5	0
**Patient N° 7**	14	77 613 858	79 875 300	2 261 442	331	2	1
**Patient N° 7**	14	80 915 590	84 405 596	3 490 006	433	5	1
**Patient N° 7**	14	85 574 003	92 332 722	6 758 719	851	5	1
**Patient N° 7**	14	92 333 611	94 458 513	2 124 902	340	2	1
**Patient N° 7**	15	32 068 300	33 795 950	1 727 650	264	2	1
**Patient N° 7**	15	33 797 342	36 142 420	2 345 078	349	5	1
**Patient N° 7**	15	84 417 316	85 512 205	1 094 889	92	2	1
**Patient N° 7**	17	46 123 698	50 671 032	4 547 334	489	5	1
**Patient N° 7**	17	51 703 484	55 181 938	3 478 454	405	3	1
**Patient N° 7**	17	55 184 769	64 453 228	9 268 459	707	4	1
**Patient N° 7**	17	64 947 310	71 177 139	6 229 829	885	4	1
**Patient N° 7**	17	71 734 008	75 110 034	3 376 026	415	5	1
**Patient N° 7**	17	75 245 211	76 442 641	1 197 430	159	3	1
**Patient N° 7**	18	26 997 913	34 682 970	7 685 057	774	5	1
**Patient N° 7**	18	35 282 864	38 729 426	3 446 562	335	3	1
**Patient N° 7**	18	44 083 807	47 269 694	3 185 887	476	5	1
**Patient N° 7**	18	48 350 316	53 744 545	5 394 229	599	5	1
**Patient N° 7**	18	53 928 436	55 513 044	1 584 608	206	0	1
**Patient N° 7**	18	55 929 965	58 177 124	2 247 159	410	3	1

**Patient N° 7**	20	37 517 986	38 628 316	1 110 330	114	2	1

[a] N. missing: number of SNPs with missing genotype; [b] heterozygotes: number of SNPs with heterozygous genotype.
