# Supplementary material for: Exome sequencing in schizophrenic patients with high levels of homozygosity identifies novel and extremely rare mutations in the GABA/glutamatergic pathways
Source: PLoS One. 2017 Aug 7;12(8):e0182778. doi: 10.1371/journal.pone.0182778 (PMC5546675; doi:10.1371/journal.pone.0182778)
Supplement: S2 Table — (PDF) [file pone.0182778.s002.pdf]

S2 Table. Exome sequencing results

Sample	Raw sequences (Gb)[a]	Mapped reads (M)[b]	Mean coverage[c]	% of bases covered at least 20X	Variants	SNPs	Indels	Ti/Tv[d]
Patient N° 1	3.6	23.4	56.8	77.4	47 955	45 182	3 247	2.57
Patient N° 2	4.4	28.1	70.7	82.1	47 739	44 276	3 839	2.60
Patient N° 3	5.3	34.9	82.4	86.0	50 655	47 902	3 079	2.54
Patient N° 4	6.9	45.7	108.0	92.9	51 628	48 879	3 155	2.50
Patient N° 5	6.9	46.2	106.5	91.1	50 848	48 104	3 091	2.49
Patient N° 6	6.0	39.2	94.7	90.2	50 642	48 046	2 966	2.52
Patient N° 7	9.2	53.9	148.4	94.4	51 553	48 760	3 166	2.55

[a] total bases sequenced;

[b] number of sequencing reads aligning to the reference genome;

[c] mean depth of coverage across the target region;

[d] transitions over Transvertions ratio.
