# Supplementary material for: Exome sequencing in schizophrenic patients with high levels of homozygosity identifies novel and extremely rare mutations in the GABA/glutamatergic pathways
Source: PLoS One. 2017 Aug 7;12(8):e0182778. doi: 10.1371/journal.pone.0182778 (PMC5546675; doi:10.1371/journal.pone.0182778)
Supplement: S3 Table — (PDF) [file pone.0182778.s003.pdf]

S3 Table. LFF and LFF-D gene lists

Sample	LFF genes [a]	LFF-D genes
Patients N° 1	*ADIPOQ, CHRM3, HDAC10, KRTAP5-3, MIEF1, OR2F2, OXER1, PRKD3, PRR34, REPIN1, SCUBE1, SULT6B1, TPRG1, UBN2, ZNF282*	*SULT6B1, OXER1, PRR34, TPRG1, UBN2, PRKD3, MIEF1*
Patients N° 2	*AASDH, ADAM29, AFP, AGA, C12orf10,* ***CIC****, CLOCK,* ***FCGBP****, FUCA2,* ***HERC1****,* ***HIVEP1****, IGDCC4, IL22RA2, IL23R,* ***KRT82****,* ***LRP1****,* ***MED29****, MEGF8, METTL21B, MMP19, MYO1A, PTGFR, SH3RF1, TESPA1, ZNF573*	***KRT82****, C12orf10, IL23R,* ***LRP1****, MYO1A,* ***HERC1****, ZNF573,* ***MED29****, MEGF8, CLOCK, AFP, SH3RF1, IL22RA2,* ***HIVEP1***
Patients N° 3	*ANO2, CDKN2A,* ***GPRIN1****, IFNA1, IFNA10, IFNA16, METTL24, MICAL1, MRVI1,* ***NCOA2****, PCSK5, PIK3CD, PRAMEF1,* ***PREX2****, SETBP1, ZSCAN30*	*ANO2, ZSCAN30, MICAL1, PIK3CD*
Patients N° 4	*ART1, HEATR5A, OR51T1, OR52N5, PHLPP1, PNLIPRP3, XRN1*	*ART1, OR51T1, XRN1*
Patients N° 5	*ATP10B, C1QTNF9B, ERMARD,* ***FAT2****, FHL5, MAP3K19, MDN1, NOD1, OVGP1, PCDH12,* ***PCDHA4****, PCDHB10, PCDHGA1, PCDHGA10, PTPN4,* ***REV3L****, RNF217, SPATA13, TCOF1, ZADH2, ZNF19*	*OVGP1, PTGFRN, ZNF19, MAP3K19, PCDHGA1, ATP10B, FHL5,* ***REV3L****, C1QTNF9B*
Patients N° 6	*ADAMTS7, ESRP1,* ***FMNL2****, GAD1,* ***MUC5B****, PHRF1, PKHD1L1, RCCD1, SLC26A7*	*GAD1, SLC26A7*
Patients N° 7	*AP5Z1, EMC7, EML5,* ***FBXL18****, FMN1,* ***FSCN1****, NACA2, NCAPD3, NDFIP2, PRCD, QRICH2,* ***SPTB****, TTYH2, UTP18*	*TTYH2, PRCD,* ***FSCN1****,* ***FBXL18****, NACA2, FMN1*

[a]Genes included also in the SZ-composite set are reported in bold. Genes expressed in Brain according to the to the EBI Expression Atlas database (FPKM > 1) [11] are underlined
