# Supplementary material for: Exome sequencing in schizophrenic patients with high levels of homozygosity identifies novel and extremely rare mutations in the GABA/glutamatergic pathways
Source: PLoS One. 2017 Aug 7;12(8):e0182778. doi: 10.1371/journal.pone.0182778 (PMC5546675; doi:10.1371/journal.pone.0182778)
Supplement: S1 Fig — The circular representation shows the distribution of ROHs (black blocks) in the seven ROH-individuals, which are reported in ascending order from the external circle as white/gray tracks. Green tracks show the genomic localization of genes/genomic regions already associated with schizophrenia in previous studies. From the external circle: SZ-GWAS set, SZ-composite set, genes from the SZGR database. Black marks on chromosome ideograms represent loci shared between schizophrenia genes/regions and our ROHs. (PDF) [file pone.0182778.s004.pdf]

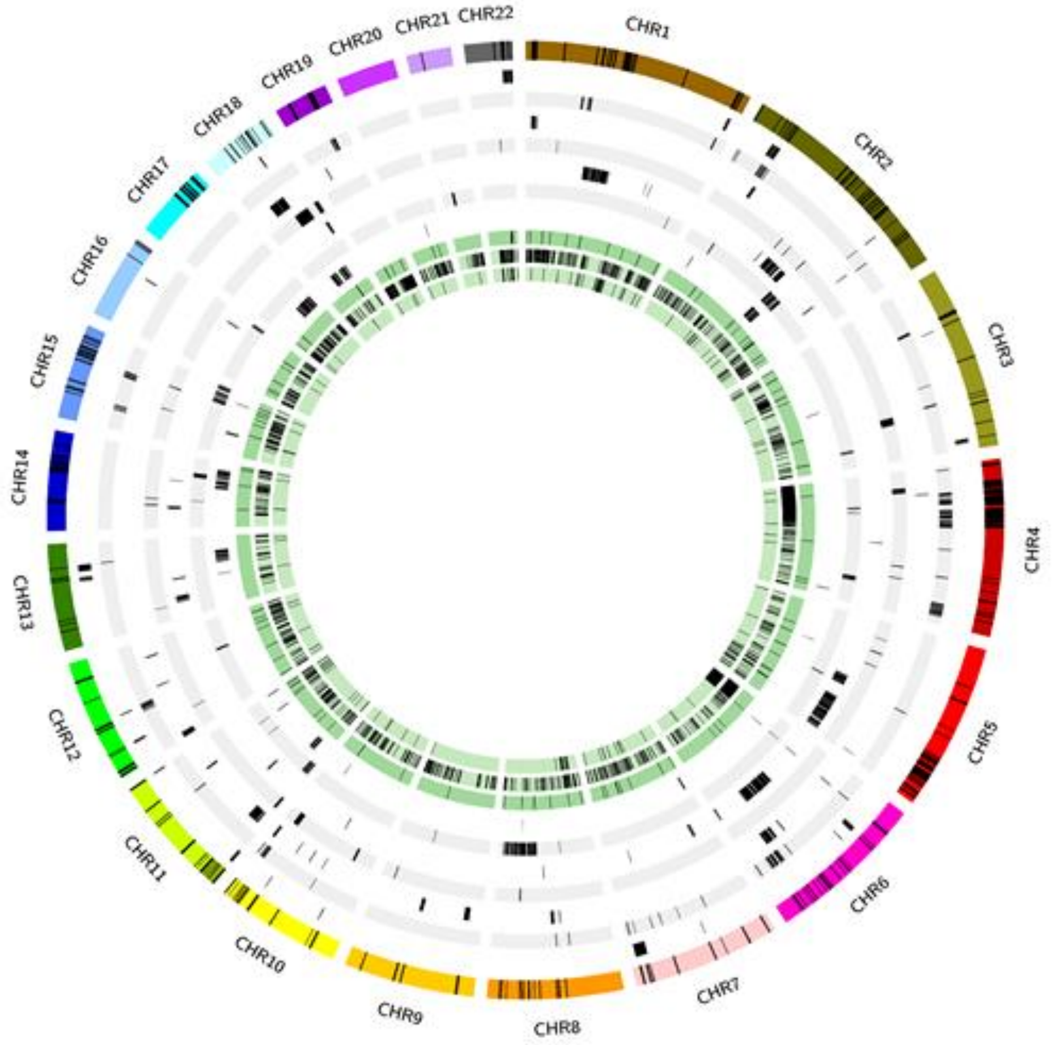

S1 Fig. ROH region distribution across the analyzed subjects. The circular representation shows the distribution of ROH regions (black blocks) in the seven ROH-individuals, which are reported in ascending order from the external circle as white/gray tracks. Green tracks show the genomic localization of genes/genomic regions already associated with schizophrenia in previous studies. From the external circle: SZ-GWAS set, SZ-composite set, genes from SZGR database. Black marks on chromosomes ideograms represent loci shared between schizophrenia genes/regions and our ROH region.
