# Supplementary material for: Exome sequencing in schizophrenic patients with high levels of homozygosity identifies novel and extremely rare mutations in the GABA/glutamatergic pathways
Source: PLoS One. 2017 Aug 7;12(8):e0182778. doi: 10.1371/journal.pone.0182778 (PMC5546675; doi:10.1371/journal.pone.0182778)
Supplement: S1 Appendix — (PDF) [file pone.0182778.s005.pdf]

S1 Appendix

Table Of Contents

Supplementary Methods **2**

Homozygosity analysis 2

Whole exome sequencing 2

Definition of candidate low frequency functional (LFF) variants in ROH regions 3

Gene Set Enrichment analysis 4

Clinical and Demographic description of ROH-individuals **4**

References **6**

Supplementary Methods

Homozygosity analysis

Detection of ROHs was performed using with SVS.7 software (Golden Helix). After pruning for strong Linkage Disequilibrium (LD) (removing any SNP with a multiple r2 > 0.90 with all others in a 50 SNP window), the ROH regions were identified, provided that they contained at least 65 consecutive homozygous SNPs with no more than one heterozygous and five missing SNPs. Finally, to ensure that ROH did not cross a region of low SNP density (e.g., a centromere), a density greater than 1 SNP per 200 kb was also required, and the ROHs were split into two parts when the gap between adjacent homozygous SNPs was greater than 500 kb. ROHs larger than 4 Mb are indicative of autozygosity due to recent parental relatedness (1, 2). Therefore, to select patients with ROHs likely due to recent inbreeding, we first selected those with at least one ROH larger than 4 Mb, and from those individuals, we selected those with the highest number of large ROHs (subjects falling in the upper quartile). In these patients, rare homozygous mutations were identified by WES in all ROH regions of at least 1Mb. (see S1 Table).

Whole exome sequencing

In order to investigate the contribution of rare variants in ROH-individuals, a WES was performed on their DNA. Sequencing libraries were prepared from 100 ng of genomic DNA using the AmpliSeq Exome kit (Life Technologies) which performs simultaneous amplifications by ultra-multiplex PCR of almost all the RefSeq CDS sequences. The final target region of ~57 Mb includes ~98% of the coding portions of RefSeq exons plus flanking regions. After digestion of PCR primers, Ion Xpress Barcode Adapters were ligated to generate the final sequencing library of each sample. Libraries were amplified with 7 PCR cycles using Platinum PCR SuperMix High Fidelity (Life Technologies). Agencourt

AMPure XP beads (Beckman Coulter Genomics) were used to purify the DNA after each reaction step. Quantification and size distribution of the amplified sequencing libraries were determined using an Agilent Bioanalyzer 2100 with the High Sensitivity DNA Kit (Agilent). After dilution to 100 pM, two exome libraries were combined and sequenced with v3 chemistry using a single Ion PI Chip v2 on a Ion Proton System (Life Technologies) according to the manufacturer's protocol.

Definition of candidate low frequency functional (LFF) variants in ROH regions

For each ROH-individual, the homozygous variants mapping inside the ROH regions reported in S1 Table were selected. A list of low frequency functional (LFF) variants was compiled for each subject. In order to be part of the list, the LFF variants had to satisfy the following criteria: (i) located in coding regions and splice sites; (ii) nonsynonymous; (iii) an allele frequency lower than 5% in 1000G, ESP6500 and ExAc 0.2 datasets. The genes containing these variants were defined as LFF genes. Among LFF variants, the missense substitutions predicted to be either damaging or possibly damaging by at least 2 out of 3 algorithms among PolyPhen2 (3), SIFT and MutationTaster (4) were selected. These variants, together with loss of function and splicing mutations, represent a list of highly probable deleterious variants (LFF-D variants) that could impair the function of the affected genes (LFF-D genes). Moreover, in order to identify the variants with the greatest impact on the schizophrenia phenotype, "best candidate" variants were selected among LFF-D variants according to the following stricter criteria: (i) predicted to be damaging by at least 2 out of 3 algorithms (ii) not reported in the dbSNP138 and with a minor allele frequency lower than 1% in the 1000G, ESP6500 or ExAc 0.2 datasets; (iii) not located in a region affected by reported segmental duplication; (iv) reported in evolutionary conserved regions (PhyloP score >0.95 or not reported). All "Best candidate" variants were visually verified in the BAM file using IGV (5) and confirmed by Sanger sequencing.

Gene Set Enrichment analysis

INRICH software was used for the gene set enrichment analyses of genes included in ROH regions over KEGG pathways, Gene Ontology (GO) categories and genes already reported to be associated with schizophrenia. The 1826 genes included in the composite set of Purcell (SZ-composite set) (6) and the 348 genes included in the 108 loci found to be associated with schizophrenia by Ripke et al. (SZ-GWAS set) (7) were considered as genes associated with the disorder.

Web GESTALT (8) was used to test the gene set enrichment of KEGG pathways and GO categories in LFF and LFF-D genes. For LFF and LFF-D genes, the list of sequenced genes (i.e. genes included in AmpliSeq Exome design) within ROH regions was considered as the background dataset.

Finally, using random permutation, the significance of the enrichment observed in LFF or LFF-D genes compared to genes already reported to be associated with schizophrenia was verified. Ten thousand permutations were performed. Each time, from the 4874 genes included in the Ampliseq Exome design and mapping inside our ROH regions, we randomly sampled a number of genes that was equal to the number of LFF and LFF-D genes, respectively, and we measured among these genes the number of genes of the SZ-composite set and of the SZ-GWAS. The enrichment p-value was then calculated as the number of times a number of the SZ-composite set and SZ-GWAS set genes equal or higher than the observed ones was observed in the permuted dataset.

Clinical and Demographic description of ROH-individuals

Patient N°1: Male, 36 years old. At 22 years old, he had the onset of psychosis. He has no psychiatric disorders in comorbidity whereas he suffers from hypertriglyceridemia and hypertransaminasemia. He has second-degree relatives affected by schizophrenia.

Patient N°2: Male, 52 years old. At 22 years old he had the onset of psychosis. He has moderate mental retardation, and nicotine and alcohol abuse as psychiatric disorders in comorbidity. He also has chronic obstructive pulmonary disease, hypercholesterolemia, diabetes mellitus, iron deficiency anemia, and pulmonary nodule tuberculosis.

Patient N°3: Male, 48 years old. At 27 years old he had the onset of psychosis. He has been diagnosed with alcohol abuse and gambling as psychiatric disorders in comorbidity, he also has hypertension, obesity, diabetes mellitus and he had a previous myocardial infarction. He has a second-degree relative affected by schizophrenia and a first-degree relative with alcohol dependence disorder. He died recently of pancreatic cancer.

Patient N°4: Female, 38 years old. At 21 years old she had the onset of psychosis. She has no psychiatric and physical disorders in comorbidity. She has a first and a second-degree relative who suffered from postpartum depression with psychotic features.

Patient N°5: Female, 42 years old. At 41 years old she had the onset of psychosis. She has moderate mental retardation as psychiatric disorders in comorbidity and she suffers from iron deficiency anemia, diabetes mellitus and recurrent headaches.

Patient N°6: The patient is a 42 year old male. At 24 years old he had the onset of psychosis. He has no psychiatric disorders in comorbidity. He has a first-degree relative, a brother, affected by schizophrenia, and a first-degree relative affected by alcohol dependence disorder.

Patient N°7: Female, 23 years old. At 23 years old she had the onset of psychosis. She has no psychiatric disorders in comorbidity but she has been diagnosed with substance abuse. She has second-degree relatives affected by schizophrenia.

References

1. McQuillan R, Leutenegger AL, Abdel-Rahman R, Franklin CS, Pericic M, Barac-Lauc L, et al. Runs of homozygosity in European populations. Am J Hum Genet. 2008;83(3):359-72.

2. Pemberton TJ, Absher D, Feldman MW, Myers RM, Rosenberg NA, Li JZ. Genomic patterns of homozygosity in worldwide human populations. Am J Hum Genet. 2012;91(2):275-92.

3. Adzhubei IA, Schmidt S, Peshkin L, Ramensky VE, Gerasimova A, Bork P, et al. A method and server for predicting damaging missense mutations. Nat Methods. 2010;7(4):248-9.

4. Schwarz JM, Cooper DN, Schuelke M, Seelow D. MutationTaster2: mutation prediction for the deep-sequencing age. Nat Methods. 2014;11(4):361-2.

5. Hott M, Marie PJ. Carbonic anhydrase activity in fetal rat bone resorbing cells: inhibition by acetazolamide infusion. J Dev Physiol. 1989;12(5):277-81.

6. Purcell SM, Moran JL, Fromer M, Ruderfer D, Solovieff N, Roussos P, et al. A polygenic burden of rare disruptive mutations in schizophrenia. Nature. 2014;506(7487):185-90.

7. Ripke S, Neale BM, Corvin A, Walters JT, Farh KH, Holmans PA, et al. Biological insights from 108 schizophrenia-associated genetic loci. Nature. 2014;511(7510):421-7.

8. Wang J, Duncan D, Shi Z, Zhang B. WEB-based GEne SeT AnaLysis Toolkit (WebGestalt): update 2013. Nucleic Acids Res. 2013;41(Web Server issue):W77-83.
